# Artificial Intelligence in Cardiac Surgery: From Assistance to Assurance

**DOI:** 10.1590/1516-3180.2026.1442.09122025

**Published:** 2026-04-10

**Authors:** Guilherme Machado Rabello, Paulo Manuel Pêgo-Fernandes, Fabio Biscegli Jatene

**Affiliations:** I Escola Politécnica da Universidade de São Paulo. Innovation Director of InovaInCor, Instituto do Coração, Hospital das Clínicas, Faculdade de Medicina, Universidade de São Paulo (USP), São Paulo (SP), Brazil.; II Thoracic Surgery Program, Instituto do Coração, Hospital das Clínicas HCFMUSP, Faculdade de Medicina, Universidade de São Paulo, São Paulo (SP), Brazil; Director, Scientific Department, Associação Paulista de Medicina (APM), São Paulo (SP), Brazil.; III Cardiovascular Surgery Division, Coordinator of InovaInCor, Instituto do Coração, Hospital das Clínicas HCFMUSP, Faculdade de Medicina, Universidade de São Paulo, São Paulo (SP), Brazil.

Artificial intelligence (AI) has evolved from a futuristic concept into an essential clinical partner. In cardiac surgery—where decisions are made in millimeters and milliseconds—AI does not threaten surgical craftsmanship but strengthens it. Algorithms capable of learning from thousands of procedures now assist surgeons with preoperative risk assessment, intraoperative precision, and postoperative surveillance.^1,2,3^ The number of AI-enabled medical devices cleared by the U.S. Food and Drug Administration has risen from 27 in 2017 to more than 1,200 in 2024, illustrating the unprecedented velocity with which digital intelligence is entering the operating room.^4^

The preoperative applications of AI are perhaps the most mature. Machine-learning models trained on multimodal datasets, from imaging to genomics, offer superior predictions of mortality, bleeding, and arrhythmic events compared with conventional risk scores. They help tailor patient selection, anticipate complications, and personalize procedural planning. Intraoperatively, AI-augmented robotics, automated perfusion control, and computer vision systems assist surgeons in maintaining accuracy and situational awareness even in complex and prolonged interventions.^5,6^ In the postoperative phase, wearable sensors and predictive analytics enable real-time surveillance and early intervention, improving recovery trajectories and reducing readmissions.^7^

At the Instituto do Coração (InCor) in São Paulo, the TRAdA Project (Telemonitoramento Remoto Assistido de Arritmia) demonstrates this transition from episodic to continuous care. By integrating AI-driven wearable devices with clinical dashboards, clinicians can detect arrhythmic events earlier, such as supraventricular tachycardia or postoperative atrial fibrillation, prompting a timely anticoagulation or ablation. These examples demonstrate that AI is not confined to the operating table; it extends the surgeon’s reach into the patient’s home, creating a loop of connected care that redefines patient follow-up and safety.^8^

However, the potency of AI depends on how institutions and professionals adapt. The challenge is not technological scarcity but responsible integration. Data quality, interoperability, and algorithmic transparency are the essential prerequisites. Bias in datasets can propagate inequity while a lack of validation can erode trust. The safe implementation of AI requires governance frameworks, multidisciplinary ethics boards, and collaboration among engineers, clinicians, and regulators. Training surgeons in data literacy and the critical interpretation of AI outputs is now as vital as learning new surgical techniques.^7^

Ethical and societal dimensions also require attention. Medical institutions and professionals must inform their patients when and how AI contributes to their care, obtain their informed consent, and maintain the accountability. Surgeon’s judgment, grounded in empathy, contextual reasoning, and experience, remains irreplaceable. AI should enhance this judgment rather than automate it. As the World Health Organization emphasizes, digital health must be human-centered and designed to strengthen, not substitute, professional expertise and compassion.

The convergence of surgery and AI marks a transformative era.^4^ When aligned with ethical standards, continuous validation, and institutional vision, AI can deliver safer operations, optimize resources, and democratize access to specialized care. It can turn cardiac surgery into a living laboratory of augmented intelligence, where innovation serves humanity.^1^ The future operating room will not be defined by who holds the scalpel, but by how intelligently the team—human and machine—thinks together.
